# Letter to the editor ‘Pharmacologic prevention and therapy of postoperative paralytic ileus after gastrointestinal cancer surgery – systematic review and meta-analysis’

**DOI:** 10.1097/JS9.0000000000001737

**Published:** 2024-06-04

**Authors:** Xinya Zhao, Biao Zhang, Xufeng Tao, Deshi Dong

**Affiliations:** a Department of Pharmacy, The First Affiliated Hospital of Dalian Medical University; b Pancreas and Biliary Center, Department of General Surgery, Clinical Laboratory of Integrative Medicine, The First Affiliated Hospital of Dalian Medical University; c College of Pharmacy, Dalian Medical University, Dalian, Liaoning, People’s Republic of China

*Dear Editor,*

We are intrigued by the recent publication in the *International Journal of Surgery* by Reichert *et al*.^1^, titled ‘Pharmacologic prevention and therapy of postoperative paralytic ileus after gastrointestinal cancer surgery – systematic review and meta-analysis’. This study conducts a systematic review and meta-analysis on the pharmacological prevention and treatment of postoperative paralytic ileus following gastrointestinal cancer surgery, offering valuable insights to guide clinical decisions and shape future research directions. However, there are several issues within this study that warrant attention and resolution.

This study encompasses a wide range of research efforts, but it does not provide detailed explanations regarding the geographic origins of these studies. Given the potential physiological differences in drug metabolism among individuals of different races, as well as variations in healthcare systems, standards, and practices across countries and regions, it is imperative to conduct further subgroup analyses of the research data^2^. This detailed analysis is crucial to ensuring the applicability of study findings to clinical patient management in diverse geographical settings and provides important reference points for personalized medical decision-making.

The data analyzed in this study is sourced from patients undergoing resection surgeries for gastrointestinal tumors, including procedures such as esophageal, gastric, pancreatic, hepatic, small intestine, and/or colorectal resections. It’s worth noting that hepatic and pancreatic resections, which do not involve reconstruction of the digestive tract, have a lesser impact on postoperative ileus (POI). Hence, this raises the question of whether these should be discussed separately or potentially excluded entirely from the analysis. These considerations warrant further discussion to determine if such an analytical approach would render the study more robust and contextually relevant.

In this study, the authors meticulously examined the existing evidence from randomized controlled trials. We believe that pooling this data for a meta-analysis is warranted. By synthesizing these findings, we can more accurately assess the overall incidence of POI in patients, a crucial determinant in guiding clinical decision-making. This comprehensive analysis empowers clinicians to better evaluate surgical risks and tailor preventive and treatment strategies accordingly, ultimately enhancing patients’ postoperative recovery and quality of life.

In summary, we commend the authors for their significant contributions to this field, as they have provided a meaningful synthesis of evidence, offering profound insights into pharmacological interventions for preventing postoperative gastrointestinal dysmotility or treating POI following gastrointestinal tumor resection. Simultaneously, we look forward to further discussions in the future to foster our collective understanding and enhance the management of POI, thereby providing crucial guidance and insights for future research and clinical practices.

## Ethical approval

Not applicable.

## Consent

Not applicable.

## Source of funding

Not applicable.

## Author contribution

X.Z. and D.D.: conceptualized the manuscript; X.Z., B.Z., X.T., and D.D.: wrote and revised the manuscript.

## Conflicts of interest diclosure

There are no competing financial interests listed by the authors.

## Research registration unique identifying number (UIN)

Not applicable.

## Guarantor

Xinya Zhao, Biao Zhang, Xufeng Tao, and Deshi Dong.

## Data availability statement

Not applicable.

## Provenance and peer review

Although our work was not by invitation, we believe that our findings will be helpful in improving the current work. Active discussion and exploration help to provide high-quality evidence-based guidance for the pharmacologic prevention and therapy of postoperative paralytic ileus of patients with gastrointestinal cancer surgery.
